# Obesity and Transcatheter Aortic Valve Replacement

**DOI:** 10.3390/jcdd11060169

**Published:** 2024-05-30

**Authors:** Jiyoung Seo, Amrin Kharawala, Pawel Borkowski, Nikita Singh, Harriet Akunor, Sanjana Nagraj, Dimitrios V. Avgerinos, Damianos G. Kokkinidis

**Affiliations:** 1 Department of Internal Medicine, Jacobi Medical Center/Albert Einstein College of Medicine, Bronx, NY 10461, USApawel.borkowski.md@gmail.com (P.B.);; 2 Department of Cardiology, Montefiore Medical Center, The University Hospital for Albert Einstein College of Medicine, Bronx, NY 10467, USA; 3 Department of Cardiac Surgery, Onassis Cardiac Surgery Center, 17674 Kallithea, Greece; 4 Section of Cardiovascular Medicine, Lawrence Memorial Hospital & Northeast Medical Group, Yale New Haven Heath, New London, CT 06614, USA

**Keywords:** obesity, transcatheter aortic valve replacement, body mass index

## Abstract

Amidst an aging population and escalating obesity prevalence, elucidating the impact of obesity on transcatheter aortic valve replacement (TAVR) outcomes becomes paramount. The so-called “obesity paradox”—a term denoting the counterintuitive association of obesity, typically a risk factor for cardiovascular diseases, with improved survival outcomes in TAVR patients relative to their leaner or normal-weight counterparts—merits rigorous examination. This review comprehensively investigates the complex relationship between obesity and the clinical outcomes associated with TAVR, with a specific focus on mortality and periprocedural complications. This study aims to deepen our understanding of obesity’s role in TAVR and the underlying mechanisms of the obesity paradox, thereby optimizing management strategies for this patient demographic, tailored to their unique physiological and metabolic profiles.

## 1. Introduction

Aortic stenosis (AS) is among the most prevalent valvular heart diseases in developed countries, with an increasing incidence reflecting the degenerative process in the aging population [1,2]. Since the first transcatheter delivery of an aortic valve prosthesis by Cribier et al. in 2002 [3], transcatheter aortic valve replacement (TAVR) was introduced as a minimally invasive alternative to surgical aortic valve replacement (SAVR) for patients with severe AS who were deemed to be at high or prohibitive surgical risk [4]. Although there are emerging issues such as the durability of transcatheter heart valves and long-term TAVR outcomes, indications for TAVR continue to expand to younger, low-risk patients with longer life expectancies [5,6]. As the utilization of TAVR expands with the annual TAVR volume in the United States, surpassing all forms of SAVR [7], it is imperative to understand the impact of diverse patient characteristics on TAVR outcomes.

The epidemiological association between obesity and an increasing incidence of various cardiovascular diseases (CVDs) such as hypertension, coronary heart disease, heart failure, and atrial fibrillation is well established [8,9,10]. Considering the obesity epidemic [11] and its growing prevalence in TAVR recipients [12], obesity has emerged as a critical factor in prognostication of clinical outcomes of TAVR. Many studies, however, have paradoxically demonstrated good prognosis and survival among obese patients than their leaner counterparts in heart failure [13,14], atrial fibrillation, and coronary heart disease [15,16], despite having established CVDs [8,17]. This phenomenon, so called the “obesity paradox”, is further applicable to the TAVR patient population [7,18,19].

We previously conducted a systematic review and meta-analysis to further explore the relationship between baseline obesity and mortality as well as the periprocedural outcomes after TAVR [20]. Here, we present an updated, comprehensive review that integrates the most recent and pertinent studies on this topic to the best of our knowledge.

## 2. Clinical Outcomes in Individuals with Overweight or Obesity Undergoing TAVR

Numerous investigations have been carried out to explore the relationship between being overweight or obese and the outcomes following a TAVR procedure. A summary of these studies, particularly focusing on mortality, peri-procedural, and postprocedural results including major bleeding, major vascular complications, cerebrovascular events, myocardial infarction, atrial fibrillation, pacemaker insertion, and acute kidney injury is presented in Table A1.

### 2.1. Mortality

The analysis of mortality outcomes in patients with overweight and obesity undergoing TAVR reveals a generally favorable trend, although some studies reported insignificant association (Table A1). The Placement of Aortic Transcatheter Valves (PARTNER) trial demonstrated a favorable correlation between a higher body mass index (BMI) and 2-year all-cause post-TAVR mortality (hazard ratio (HR) per BMI unit increase 0.95; 95% confidence interval (CI) 0.91–0.98; *p* = 0.005) [21,22]. Additionally, a comprehensive cohort study examining the Transcatheter Valve Therapy (TVT) registry indicated improved 1-year all-cause mortality rates in overweight (BMI, 25.0–29.9 kg/m^2^), class I (BMI, 30.0–34.9 kg/m^2^) and class II (BMI, 35.0–39.9 kg/m^2^) obesity compared to normal-weight patients (HR 0.88, 95%CI 0.81–0.95; HR 0.8, 95%CI 0.72–0.90; HR 0.84, 95%CI 0.72–0.98, respectively) [23]. This observation led to the hypothesis that a lower BMI might be indicative of greater frailty, thereby yielding a poorer prognosis compared to counterparts with a higher BMI. Notably, De Palma et al. [18] expanded this understanding by assessing not just the absolute weight at the time of TAVR but also considering the trajectory of weight change before the procedure as a crucial determinant of patient outcomes. In this study, patients with BMI > 25 kg/m^2^ at the time of TAVR manifested a lower 3-year all-cause mortality compared to their counterparts with BMI < 25 kg/m^2^ (HR 0.68, 95%CI 0.5–0.93, *p* = 0.02), consistent with the obesity paradox. However, this paradigm shifts for patients in the overweight (BMI 25.1–30 kg/m^2^) and obese (BMI 30.1–35 kg/m^2^) categories who had experienced significant weight loss leading to a change in BMI classification, with poorer 1-year mortality outcomes compared to those with stable weight trajectories (HR 1.64, 95%CI 1.06–2.3, *p* = 0.025). Interestingly, the BMI < 25 kg/m^2^ group had better 3-year mortality outcome when compared to the severe obesity group (BMI > 35 kg/m^2^) (HR 1.64, 95%CI 1.06–2.3, *p* = 0.025), and those who gained weight pre-TAVR had a higher 1-year mortality risk than those without weight change, although it was statistically insignificant (HR 2.1, 95%CI 0.96–4.0, *p* = 0.062). This represents intricate interplay between obesity and TAVR outcomes that necessitates further research to better understand their complex interaction.

### 2.2. Periprocedural Complications

#### 2.2.1. Major Vascular Complications

Obesity has been identified as an independent risk factor for vascular complications in patients undergoing TAVR [24,25,26]. The existing literature indicates that the incidence of vascular complications in this demographic ranges from 10% to 16% [27,28,29]. Our prior research corroborates this, revealing higher odds of major vascular complications (OR: 1.33; 95%CI: 1.05–1.68) in patients with obesity [20]. While the transfemoral (TF) approach is predominantly utilized in TAVR procedures due to its minimally invasive nature, the management of femoral vascular access and hemostasis in patients with obesity is challenging due to their unique anatomical characteristics. The common femoral artery in such individuals can be up to 10 cm beneath the skin surface [30,31], complicating its access. This complexity is compounded by the challenges in determining the appropriate gauge angulation and securing optimal manual compression proximal to the puncture site, thereby escalating the likelihood of vascular complications [26]. Consequently, vigilance monitoring is essential when attempting vascular access in obese patients to mitigate these increased risks [26,32]. Alternative vascular access routes, such as transcarotid or trans-subclavian approaches, are being explored in this context [26,33,34].

Transcarotid (TC) access for TAVR offers several advantages, particularly in obese patients, being superficial, closer to the skin, and of a shorter distance to the aortic annulus, ultimately facilitating procedural maneuvers [26]. Compared to more invasive transapical and transaortic approaches, studies have shown that the safety and efficacy outcomes of TC-TAVR can parallel those of TF-TAVR in real-world populations [35,36,37]. TC access has been linked to a low rate of vascular and bleeding complications, which is beneficial for TAVR candidates with significant obesity [26,37]. Though there are concerns about cerebrovascular complications, recent studies report stroke rates comparable to the TF approach [37,38,39], although there remains a lack of definitive evidence on the superiority of any non-TF access sites [33].

The trans-subclavian (TS) approach is another emerging alternative to the transfemoral approach, which has yielded similar early mortality, stroke, and major complication rates to the TC access in a general TAVR population [26,34]. Compared to transapical and transaortic TAVR, which require chest entry and have demonstrated worse outcomes, these new methods are considered less invasive and potentially more suitable for specific patient cohorts with severe femoral atherosclerosis, body habitus issues precluding femoral access, or lung diseases, which are common in morbidly obese individuals [40,41,42,43]. However, specific data regarding obese TAVR recipients are limited, and further validation through large-scale registries and randomized trials are essential to confirm these findings, particularly in the obese population who are ineligible for traditional approaches.

#### 2.2.2. Pacemaker

Pacemaker (PPM) implantation due to conduction abnormalities post-TAVR is a significant complication, with its incidence rate reported to range from 5% to 33% [44,45,46,47,48,49]. The data from the PARTNER trial identified chronic pacing as an independent factor predicting 1-year mortality post-TAVR [50,51]. Furthermore, PPM implantation is associated with increased overall healthcare costs and prolonged hospitalization [50,51,52,53,54]. The etiology of an atrioventricular (AV) conduction block following TAVR is hypothesized to be related to direct injury to the bundle of His, owing to its proximity to the membranous septum and native aortic valve [52,55]. Other factors, including a pre-existing right bundle branch block (RBBB), the implantation depth, the use of a self-expanding valve, prosthesis-to-left-ventricular-outflow-tract-diameter ratio, male gender, a prolonged partial response interval, and a left anterior hemiblock, have also been identified as predictors of a PPM implantation [45,50,52,56,57,58]. The association between BMI and the necessity for a PPM implantation post-TAVR, however, has not been extensively explored. A retrospective study of 449 patients undergoing TAVR found a significant association between increased BMI (>30 kg/m^2^) and the need for PPM implantation (*p* = 0.037), though potential independent confounders related to BMI were acknowledged [52]. A meta-analysis involving 981,168 post-TAVR patients across 239 studies identified BMI over 25 kg/m^2^ as a weak predictor for PPM implantation (risk ratio 1.08; *p* = 0.05) [45]. Obesity may contribute to metaplastic and infiltrative changes in the sinus node, AV node, right bundle branch, and myocardium adjacent to the AV ring, leading to cardiac conduction anomalies [52,59,60]. However, the extent to which these alterations predispose obese patients to post-TAVR PPM implantation remains unclear, underscoring the need for further investigation in this area.

#### 2.2.3. Acute Kidney Injury

The Valve Academic Research Consortium (VARC) recognizes renal impairment as a major outcome parameter following TAVR [61]. Factors contributing to acute kidney injury (AKI) post-TAVR include periprocedural hypotension [62], blood loss [63], concurrent medication use, the administered volume of contrast agent [64], and severe inflammatory response syndrome following intervention [65,66]. Nevertheless, the existing literature on the incidence of AKI post-TAVR is not definitive, and the association between obesity and post-TAVR AKI remains varied. The research conducted by Schnabel et al. [65] identified BMI as a significant predictor of postprocedural renal function deterioration (β = −1.2; *p* < 0.0001). Additionally, a study by Ogami et al. [67] of 3883 patients with end-stage renal disease (ESRD) undergoing TAVR found that patients with a BMI below 25 kg/m2 had higher risks of 1-year (HR: 1.2; 95%CI: 1.07–1.36; *p* = 0.003) and 5-year mortality (HR: 1.18; 95%CI: 1.08–1.29; *p* < 0.001) compared to those with a BMI above 25 kg/m^2^. While these findings might suggest a protective effect of a higher BMI on renal function post-TAVR, in line with the ‘obesity paradox’, the underlying mechanism remains uncertain. Conversely, other studies report no significant or even adverse associations between a high BMI and renal outcomes following TAVR. A study from the PARTNER 1 trial, comparing groups based on changes in estimated glomerular filtration rate (eGFR) post-TAVR—improved, worsened, or unchanged—found no significant differences in the BMI among these cohorts [68]. Additionally, Koifman et al. [69] identified an association between a higher BMI and an increased incidence of AKI in TAVR patients, as determined using Risk Injury Failure Loss End-Stage (RIFLE) criteria (OR 1.12; *p* = 0.01). It is crucial to note that the eGFR equation does not incorporate any measure of body size and is subject to inherent biases in different patient groups, particularly in relation to age, gender, and BMI [70]. Furthermore, factors such as fluid status changes may more significantly influence eGFR than serum creatinine levels [69,70]. Consequently, there is a pressing need for further research in this field to elucidate these complex relationships and refine diagnostic and prognostic criteria for renal function assessment post-TAVR.

#### 2.2.4. Miscellaneous

The association between obesity and other post-TAVR complications, including cerebrovascular events, new-onset atrial fibrillation, and myocardial infarction has received limited research attention.

## 3. Exploring Potential Mechanisms of the Obesity Paradox

The counterintuitive impact of BMI on clinical outcomes may be attributed to several potential mechanisms. One such mechanism is the increase in total blood volume and cardiac output in individuals with obesity, primarily due to the increased metabolic demands associated with excess body weight [59,60,71]. This elevation in cardiac output and stroke volume may provide an enhanced hemodynamic reserve, enabling these patients to better tolerate procedural stress. Furthermore, adipose tissue is found to synthesize tumor necrosis factor-alpha (TNF-α) receptors [27,72]. Therefore, individuals with overweight and obesity might have a protective adaptation against the harmful effects of elevated TNF-α levels through increased receptor production [27]. Additionally, various studies indicate that individuals with obesity often present at a younger age, are more prone to pursue medical care promptly, and are more likely to undergo aggressive medical and interventional treatments at earlier stages [7,18,27,28,73,74,75].

The obesity paradox is primarily pronounced in older individuals who typically exhibit more symptoms, poorer ventricular function, and a higher burden of comorbidities. The pathophysiology of this phenomenon has been extensively studied in geriatric medicine and patients with heart failure. A potential mechanism is the association of undernutrition, sarcopenia, and cardiac cachexia which is commonly associated with frailty [76,77,78,79,80]. The current guidelines, American College of Cardiology (ACC) and European Society of Cardiology/European Association for Cardio-Thoracic Surgery (ESC/EACTS), recommend incorporating frailty assessments in patient selection for TAVR, as it is associated with adverse outcomes [80,81,82]. Frailty refers to a state of impaired homeostatic reserve and reduced resilience to stressors which, in turn, increases susceptibility to adverse health outcomes [83,84,85]. While there are numerous methods to assess frailty, the VARC characterizes frailty through multifactorial aspects like slowness, weakness, wasting, malnutrition, poor endurance, inactivity, and reduced independence [86].

Understanding the obesity paradox in the TAVR population requires a multidisciplinary approach, integrating insights from diverse fields such as cardiology, endocrinology, bariatric surgery, geriatrics, and nutrition science. This comprehensive strategy is essential for precisely delineating the primary etiology of the patient’s symptoms, particularly in instances where exertional dyspnea may be multifactorial, and providing individualized interventions aimed at optimizing patient TAVR outcomes.

## 4. Morbidly Obese Population: Exploring the Extremes

The incidence of obesity in the United States has escalated to near-epidemic levels, with the data from 2017 to 2018 indicating that 42% of U.S. adults meet the criteria for class I obesity (BMI ≥ 30 kg/m^2^) and 9% are classified as having severe, morbid, class III obesity (BMI ≥ 40 kg/m^2^) [87]. Morbid obesity (MO) is recognized not only as a significant cardiovascular risk [10], but also as a determinant of high surgical risk [88]. This cohort of patients, although frequently encountered in real-world clinical practice, tends to be underrepresented in clinical trials. Although the ‘obesity paradox’ is noted in patients undergoing TAVR, the precise mechanism and the extent to which it applies, especially in instances of extreme obesity, remain unclear. Hence, studies to determine the safety profile and risk factors associated with the MO population are imperative. The prevailing hypothesis suggests a J-shaped correlation between BMI and TAVR outcomes, wherein favorable results are associated with overweight and mild obesity, while underweight or morbid obesity are associated with adverse outcomes [23,89,90].

Sharma et al. [23] investigated the relationship between BMI and both short- and long-term outcomes following TAVR in a substantial cohort of 31,929 patients undergoing TAVR. Utilizing BMI as a continuous variable, their analysis revealed that for BMIs of 30 kg/m^2^ or less, each 1 kg/m^2^ increment was associated with a 2% and 4% reduction in 30-day and 1-year mortality risks, respectively. However, for BMIs over 30 kg/m^2^, each 1 kg/m^2^ increment corresponded to a 3% increased risk of 30-day mortality but not to 1-year mortality. This study supports the J-shape hypothesis with the nadir of BMI at 30 kg/m^2^ and suggests an optimal BMI for post-TAVR outcomes within the range of overweight to mild obesity.

Another study conducted by McInerney et al. [91] compared propensity matched cohorts of 770 patients with MO with equal number of nonobese patients. The study found significantly higher incidence rates of post-TAVR major vascular complications (6.6% vs. 4.3%; *p* = 0.043) and lower rates of device success (84.4% vs. 88.1%; *p* = 0.038) in the MO group. However, this did not translate into increased all-cause and cardiovascular mortality at 2 years, which remained comparable between the two groups. Similarly, Ferreiro et al. [92] found no significant difference in the in-hospital, 30-day, and 1-year (12.5% in MO vs. 15.3% in non-MO; *p* = 0.76) post-TAVR mortality when comparing 25 patients with BMI ≥ 40 kg/m^2^ vs. A total of 493 patients had a BMI < 40 kg/m^2^. In fact, no significant differences were seen even in 1-year cardiovascular mortality rate (12.5% in MO vs. 10.8% in non-MO; *p* = 0.827) and heart failure (HF) hospitalization rate (14.3% in MO vs. 16.5% in non-MO; *p* = 0.825). Interestingly, however, they also found higher trends of major vascular complications and vascular closure device failure, corroborating with the findings of our meta-analysis (20) which indicated increased vascular complications in obese cohorts, likely attributed to the challenges in transfemoral access. Of particular interest, a comparison of propensity-matched cohorts of MO patients undergoing TAVR vs. SAVR across 15 centers [93], revealed similar overall outcomes in terms of all-cause mortality, CV mortality and readmissions rates, despite differing predictors of the two-year all-cause mortality. On the contrary, the PARTNER 2A trial [94,95] yielded evidence demonstrating that among intermediate surgical risk patients (predicted 30-day surgical mortality of 4% to 8% determined by the STS mortality risk model [96]) afflicted with severe AS and BMI ≥ 35 kg/m^2^, those undergoing TAVR exhibited a statistically significant reduction in cardiovascular mortality when compared to their counterparts undergoing SAVR. Though the question remains on how MO patients fare in comparison to the overweight/mild obesity groups, the overall safety of TAVR in MO patients has been established.

These studies, however, are not without limitations. It was noted that the MO cohort in Ferreiro et al. [92] consisted of younger patients, more women, a significantly lower rate of coronary artery disease, and a lower risk profile, which could explain the lack of worse mortality outcomes. Moreover, the follow-up duration of 1–2 years in these studies might not comprehensively assess mortality outcomes. Another consideration is the definition of morbid obesity based on BMI, where factors beyond mere ‘weight’ could influence mortality outcomes and adverse effects. Several studies [10,91,97,98] utilizing imaging modalities to characterize visceral adiposity, including ectopic fat, have identified the composition and distribution of fat as an independent indicator of poor cardiovascular outcomes, irrespective of weight or BMI. Hence, further studies exploring other obesity metrics beyond BMI is imperative to effectively risk-stratify individuals with obesity undergoing TAVR [28].

## 5. Evaluating BMI as an Appropriate Surrogate for Obesity in TAVR

### 5.1. Body Mass Index

BMI is the ratio of body mass in kilograms (kg) to the square of height in meters (m^2^). The World Health Organization (WHO) stratifies BMI into various categories: underweight (BMI < 18.5 kg/m^2^), normal (BMI 18.5–24.9 kg/m^2^), overweight (BMI 25–29.9 kg/m^2^), and three classes of obesity (Class I: BMI 30–34.9 kg/m^2^, Class II: BMI 35.0–39.9 kg/m^2^, Class III: BMI ≥ 40 kg/m^2^) [99]. Historically, BMI has been a widely accepted measure of obesity. It is favored due to its simplicity in calculation, standardization, and established association with comorbid conditions such as hypertension, diabetes, and coronary artery disease, which can significantly impact TAVR outcomes. Using BMI allows clinicians to identify patients who may be at a higher risk for specific post-TAVR complications. However, BMI carries considerable limitations. It may not accurately reflect health risks across different ages, genders, and ethnicities, and it also fails to reflect health behaviors [100,101]. Furthermore, despite its association with abdominal adiposity, BMI does not differentiate between adipose and lean body mass, nor does it account for the distribution pattern of body fat (visceral adipose tissue [VAT] vs. subcutaneous adipose tissue [SAT]). This renders BMI a less precise indicator of obesity and obscures the understanding of which component of BMI influences clinical outcomes in TAVR [100,102]. The distribution of adipose tissue is of critical relevance in TAVR, affecting procedural technicalities, vascular access, device deployment, postoperative recuperation, and overall suitability for the intervention [91,103]. In the majority of previous studies investigating the ‘obesity paradox’ in the TAVR population, BMI has been the predominant obesity metrics. However, considering the intrinsic limitations of BMI, research endeavors aimed at discerning alternative adiposity metrics that may prognosticate TAVR outcomes with enhanced accuracy are necessary. Table 1 presents a summary of the obesity metrics employed in the evaluation of TAVR outcomes.

### 5.2. Alternative Obesity Metrix for TAVR Outcomes

#### 5.2.1. Body Surface Area (BSA)

BSA is deduced via a formula that incorporates both an individual’s weight (kg) and height (cm). BSA may represent a more refined index for adiposity than BMI, attributable to its capacity to more precisely reflect the disparate densities of muscle and adipose tissue [104,105,106]. There are limited studies, however, evaluating BSA as a prognostic tool for TAVR outcomes, leaving their association unclear. Watanabe et al. [107] conducted a retrospective analysis of 424 individuals undergoing TAVR and compared outcomes between groups categorized by low BSA (<1.75 m^2^) and high BSA (>1.75 m^2^). The study reported a higher incidence of vascular complications in the low BSA group (13% vs. 4.3%, *p* < 0.01), but no significant difference in mid-term survival rates (*p* = 0.64). Furthermore, a prospective observational study carried out by Arsalan et al. [105], encompassing 917 TAVR patients, sought to assess the impact of both BMI and BSA on post-TAVR outcomes. Notably, this study revealed that the “obesity paradox” phenomenon emerged only when patients were assessed using BMI with significant association with 1-year survival rates (*p* = 0.01). However, no statistically significant correlation was observed between increased survival and elevated BSA (*p* = 0.13). These findings underscore the need for continued research into BSA as a potentially valuable metrics in TAVR, offering insights distinct from BMI in the evaluation of TAVR outcomes linked to obesity.

#### 5.2.2. Abdominal Fat Analysis via Computed Tomography (CT) Scans

As BMI is a relatively crude obesity marker failing to differentiate between adipose and muscle mass, there has been growing interest in identifying obesity phenotypes, beyond mere BMI [93,108]. Numerous previous studies have underscored the significance of adipose tissue’s anatomical localization in relation to the predisposition to various diseases, and abdominal obesity is recognized as one of the critical risk factors [100,109]. Particularly, abdominal visceral adipose tissue (VAT) and subcutaneous adipose tissue (SAT) exhibit a robust correlation with the risk of CVDs and metabolic disorders [100,110,111]. The direct visualization and quantification of VAT vs. SAT using CT scans can be instrumental in assessing an individuals’ obesity phenotype and the distribution of adipose tissue components. The regular use of CT scans can expose patients to radiation which can pose a limitation for its wide-scale application. Pre-procedural CT, however, is crucial in the procedural planning of TAVR, which makes SFA and VFA easily measurable and usable as a valuable prognostic marker in TAVR candidates [102,112]. Okuno et al. [102] investigated the association between abdominal total fat area (TFA), VAT, or SAT, and TAVR outcomes in 100 individuals undergoing TAVR with a median follow-up of 665 days. The patients with higher SAT exhibited a significantly reduced incidence of the composite outcome and all-cause mortality compared to those with lower SAT (15.0% vs. 37.7%, *p* = 0.025; and 8.9% vs. 23.7%, *p* = 0.047, respectively). In contrast, the individuals with higher TFA or VAT did not demonstrate a significant reduction in the incidence of the composite outcome or all-cause mortality. Furthermore, McInerney et al. [91] identified those with a VAT:SAT ratio ≥ 1 as an adverse obesity phenotype in morbid obesity cohort, with significantly increased risks for 2-year all-cause mortality (HR: 3.06; 95%CI: 1.20–7.77; *p* = 0.019) and cardiovascular mortality (HR: 4.11; 95%CI: 1.06–15.90; *p* = 0.041). This continued to remain a strong predictor of 2-year mortality even on multivariable analysis. Visceral adipose tissue, characterized as a metabolically active ectopic fat deposit, is linked with dysregulation of lipid metabolism and insulin resistance [113]. In contrast, subcutaneous adipose tissue has been found to have cardiovascular advantages, primarily due to the secretion of adiponectin, which manifests anti-inflammatory properties, enhances insulin sensitivity, and mitigates atherogenesis [102,114]. Hence, there is a compelling need to promote future investigations that utilize visceral adiposity or adipose tissue distribution as biomarkers for discerning adverse obesity phenotypes to enhance the precision of risk stratification for obese individuals undergoing TAVR.

#### 5.2.3. Epicardial Adipose Tissue (EAT) Volume via CT Scan

EAT is a visceral fat located between the myocardium and the visceral pericardium [77,115]. It shares an embryological origin with the epicardial layer of the myocardium and visceral adipose tissue, distinct from subcutaneous adipose tissue [116]. EAT is a metabolically active tissue that has localized endocrine effects [115] and secretes pro- and anti-inflammatory mediators, including adiponectin, interleukin-6, and TNF-α [77,117,118]. Its association with CVDs, such as coronary artery disease [119,120,121], atrial fibrillation [122], and major cardiovascular events [91,110,115,123,124], has been extensively established. While various imaging modalities are capable of assessing EAT, CT is acknowledged as the most precise method for its quantification, attributed to its superior spatial resolution and comprehensive coverage of the heart [77,115]. Several research initiatives have probed the impact of EAT volume on TAVR outcomes. Eberhard et al. [77], in a study involving 503 TAVR patients, observed that a higher EAT volume was associated with increased 1-, 2-, and 3-year all-cause post-TAVR mortality (HR: 1.94, 95%CI: 1.15–3.26, *p* = 0.002; HR: 1.70, 95%CI: 1.06–2.68, *p* = 0.001; HR: 1.69, 95%CI: 1.10–2.60, *p* = 0.001, when using 100 mm^3^, 125 mm^3^, and 130 mm^3^ as cutoff values, respectively). Similarly, McInerney et al. [91] reported that each 10 cm^3^/m^2^ increment in indexed EAT was associated with an increased risk of all-cause mortality at 2 years (HR, 1.16; 95%CI, 1.03–1.30; *p* = 0.011). Notably, Eberhard’s study cohort exhibited a weak correlation between EAT volume and both BMI (BMI; r = 0.24; *p* < 0.001) and BSA (r = 0.26; *p* < 0.001), aligning with previous findings that associate EAT with coronary artery disease independent of BMI [119]. This observation prompts a question into whether the EAT volume should be linked with general obesity in the context of the ‘obesity paradox’, despite its common embryological origin with VAT. Continued exploration into the intricate influence of EAT on TAVR outcomes and its association with obesity is crucial in discerning high-risk profiles among TAVR candidates.

#### 5.2.4. Other Metrics

Alternative measures, such as the visceral adiposity index, body adiposity index, or body composition analysis (e.g., dual-energy X-ray absorptiometry and bioelectrical impedance analysis), may offer a more holistic assessment of obesity [100]. Nevertheless, their prognostic efficacy concerning TAVR outcomes warrants further research.

## 6. Conclusions

In conclusion, our comprehensive review underscores the complexity in this field, highlighting the heterogeneity in TAVR outcomes observed across varying degrees of obesity and employing different obesity metrics. The nuanced influence of obesity on TAVR outcomes necessitates deeper exploration, particularly considering the unique physiological and metabolic profiles inherent to individuals with obesity. Additionally, the question of whether TAVR outcomes can be modified by weight management interventions, including bariatric surgery, warrants further investigation. As the domain of TAVR continues to evolve, understanding the adipose tissue dynamics on cardiovascular health and the development of sophisticated obesity metrics will be crucial for a more refined risk stratification and for optimizing the management of individuals with obesity undergoing TAVR.

## Figures and Tables

**Table 1:** Obesity metrics employed in the evaluation of TAVR outcomes.

Obesity Metrics	Strengths	Limitations
BMI (Body Mass Index)	Widely used in both clinical and research settings	May not accurately reflect health risks across different ages, genders, ethnicities
	Easy to calculate and is standardized	Does not differentiate between fat and muscle mass
	Provides a general assessment of obesity	Does not account for body fat distribution (visceral adipose tissue [VAT] vs. subcutaneous adipose tissue [SAT])
	Well-established association with various comorbidities, such as hypertension and diabetes	
BSA (Body Surface Area)	Offers a more comprehensive evaluation of body size	Predictive value for TAVR outcomes is not firmly established
	Can provide insights into how a patient’s body size may impact medical outcomes	Limited evidence regarding its superiority over BMI
Abdominal Adipose Tissue via CT	Allows direct visualization and quantification of metabolically active fat deposits	Limited wide-scale application due to radiation exposure
	May offer more specific insights into fat distribution and its impact on health risks	
Epicardial Adipose Fat Volume via CT	Offers quantification of fat volume directly affecting the heart via paracrine or cytokine release	Limited representation of general obesity
		Limited wide-scale application due to radiation exposure

## Data Availability

Not applicable.

## Appendix A

**Table A1:** Summary of research findings on mortality and peri-/post-procedural TAVR outcomes in overweight/obese patients. This table categorizes study outcomes as Favorable (indicating positive outcomes in overweight/obese patients), Unfavorable (indicating negative outcomes in these patients), or Insignificant (denoting statistically non-significant results). In cases where studies presented multiple findings, outcomes derived from adjusted or multivariable models were prioritized. Procedural complications were defined by the Valve Academic Research Consortium (VARC) ([125]).

Study	Year	Country	Sample Size (*n*)	BMI Classification	Mortality	Major Bleeding	Major Vascular Complications	Cerebrovascular Events	MI	AF	PPM	AKI
Abawi et al. [27]	2017	Netherlands	549	WHO	Favorable all-cause 30-days (HR 0.69, 95%CI 0.47–0.99) and 1-year mortality (HR 0.65, 95%CI 0.45–0.94) in NW vs. OW pts	Insignificant in NW vs. OW, NW vs. O, OW vs. O	Insignificant in NW vs. OW, NW vs. O, OW vs. O	Insignificant in NW vs. OW, NW vs. O, OW vs. O	-	Insignificant in NW vs. OW, NW vs. O, OW vs. O	Insignificant in NW vs. OW, NW vs. O, OW vs. O	Insignificant in NW vs. OW, NW vs. O, OW vs. O
Abramowitz et al. [4]	2016	USA	805	WHO	Insignificant all-cause 2-year mortality in OW vs. NW (HR 1.07, 95%CI 0.67–1.71, *p* = 0.77) and O vs. NW (HR 1.03, 95%CI 0.54–1.95, *p* = 0.96)	Insignificant in NW vs. OW vs. O	Insignificant in NW vs. OW vs. O	Insignificant in NW vs. OW vs. O	Insignificant in NW vs. OW vs. O	-	Insignificant in NW vs. OW vs. O	Insignificant in NW vs. OW vs. O
Ahmad et al. [52]	2019	USA	449	UW: BMI < 25 kg/m^2^; NW: 25 kg/m^2^ ≤ BMI < 30 kg/m^2^; OW: 30 kg/m^2^ ≤ BMI < 35 kg/m^2^; O: BMI ≥ 35 kg/m^2^	-	-	-	-	-	-	Unfavorable in OW vs. NW (OR 12.77, 95%CI 1.39–17.25, *p* = 0.024) and O vs. NW (OR 15.02, 95%CI 1.19–19.92, *p* = 0.036)	-
Alharbi et al. [126]	2023	USA	77,319 TAVR hospitalizations	WHO	Insignificant inpatient mortality in UW vs. NW (OR 5.85, 95%CI 0.47–72.76, *p* = 0.16), OW vs. NW (OR 0.26, 95%CI 0.01–5.13, *p* = 0.38), class I and II obesity vs. NW (OR 0.3, 95%CI 0.02–3.66, *p* = 0.35), class III obesity vs. NW (OR 0.75, 95%CI 0.05–11.0, *p* = 0.83)	Insignificant in UW vs. NW, OW vs. NW, class I and II obesity vs. NW, class III obesity vs. NW	-	-	-	Favorable in OW vs. NW (OR 0.36, 95%CI 0.19–0.69, *p* < 0.001), class I and II obesity vs. NW (OR 0.54, 95%CI 0.32–0.91, *p* = 0.02), class III obesity vs. NW (OR 0.52, 95%CI 0.29–0.95, *p* = 0.04)		Favorable in OW vs. NW (OR 0.24, 95%CI 0.11–0.59, *p* < 0.001), class I and II obesity vs. NW (OR 0.35, 95%CI 0.18–0.71, *p* < 0.001)
Arsalan et al. [105]	2016	Germany	917	BMI used as a continuous variable	Insignificant 30-day mortality (*p* = 0.25), but Favorable 1-year mortality (*p* = 0.01)	-	Insignificant	Insignificant	-	-	-	Insignificant
Berti et al. [127]	2021	Italy	3776	WHO	-	Insignificant in OW and O vs. NW (OR 0.88, 95%CI 0.51–1.51, *p* = 0.6)	Insignificant in OW and O vs. NW (OR 1.4, 95%CI 0.94–2.07, *p* = 0.09)	-	-	-	-	-
Boukhris et al. [7]	2021	Canada	412	UW: BMI < 20 kg/m^2^; NW: 20 kg/m^2^ ≤ BMI < 25 kg/m^2^; OW: 25 kg/m^2^ ≤ BMI < 30 kg/m^2^; O: BMI ≥ 30 kg/m^2^	Insignificant 30-day [UW vs. NW (HR 1.19, 95%CI 0.14–10, *p* = 0.873), UW vs. OW (HR 1.03, 95%CI 0.11–9.19, *p* = 0.979), UW vs. O (HR 1.02, 95%CI 0.09–15.17, *p* = 0.969)] and 1-year all-cause mortality [UW vs. NW (HR 1.09, 95%CI 0.16–7.59, *p* = 0.926), UW vs. OW (HR 1.04, 95%CI 0.82–1.32, *p* = 0.707), UW vs. O (HR 1.2, 95%CI 0.59–2.42, *p* = 0.617)]	-	Insignificant in UW vs. NW vs. OW vs. O	Insignificant in UW vs. NW vs. OW vs. O	-	-	Insignificant in UW vs. NW vs. OW vs. O	Insignificant in UW vs. NW vs. OW vs. O
Corcione et al. [11]	2021	Italy	3075	WHO	Unfavorable 1-month all-cause mortality in NW vs. O (OR 0.41, 95%CI 0.18–0.95, *p* = 0.037), Favorable mid-term all-cause mortality (9.8 to 11.8 months) in O vs. UW (OR 0.5, 95%CI 0.27–0.93, *p* = 0.028)	Insignificant in UW vs. NW vs. OW vs. O	Insignificant in UW vs. NW vs. OW vs. O	Insignificant in UW vs. NW vs. OW vs. O	Insignificant in UW vs. NW vs. OW vs. O	-	Unfavorable in NW vs. OW (OR 0.76, 95%CI 0.59–0.97, *p* = 0.031) and NW vs. O (OR 0.66, 95%CI 0.49–0.9, *p* = 0.008)	Insignificant in UW vs. NW vs. OW vs. O
De Marzo et al. [12]	2021	Italy	645	UW and NW: BMI < 25 kg/m^2^; OW: 25 kg/m^2^ ≤ BMI < 30 kg/m^2^; O: BMI ≥ 30 kg/m^2^	Insignificant 30-day (*p* = 0.86), 6-month (*p* = 0.9) and 1-year (*p* = 0.31) all-cause mortality in UW and NW vs. OW vs. O Insignificant 30-day (OR 1.54, 95%CI 0.51–4.68, *p* = 0.45), 6-month (OR 1.46, 95%CI 0.55–3.94, *p* = 0.45), and 1-year (OR 1.74, 95%CI 0.98–3.07, *p* = 0.09) all-cause mortality (BMI as continuous variable)	Insignificant	Insignificant	Insignificant	Insignificant	-	Insignificant	Insignificant
De Palma et al. [18]	2018	Sweden	493	UW: BMI < 18 kg/m^2^; NW: 18 kg/m^2^ ≤ BMI < 25 kg/m^2^; OW: 25 kg/m^2^ ≤ BMI < 30 kg/m^2^; O: 30 kg/m^2^ ≤ BMI < 35 kg/m^2^; Very Obese: BMI ≥ 35 kg/m^2^	Favorable all-cause 1497-day mortality in BMI > 25 vs. BMI < 25 (HR 0.68, 95%CI 0.5–0.93, *p* = 0.02) and BMI > 25 vs. BMI < 35 (HR 0.63, 95%CI 0.45–0.86, *p* = 0.005)	-	-	-	-	-	-	-
Gilard et al. [128]	2016	France	4201	BMI categorized into <18.5, 18.5–29.9, ≥30 kg/m^2^ groups	Favorable all-cause 3-year mortality in BMI ≥ 30 vs. BMI (18.5–29.9) (HR 1.15, 95%CI 0.99–1.33, *p* = 0.08) and BMI ≥ 30 vs. BMI < 18.5 (HR 1.76, 95%CI 1.29–2.4, *p* < 0.001)	-	-	-	-	-	-	-
Gonska et al. [129]	2021	Germany	400	BMI categorized into <25 and ≥25 kg/m^2^ groups	-	-	Insignificant in BMI < 25 vs. BMI ≥ 25 (OR 0.69, 95%CI 0.41–1.19, *p* = 0.19)	-	-	-	-	-
Gonzalez-Ferreiro et al. [90]	2016	Spain	770	WHO	Favorable all-cause 3-year mortality in OW vs. NW (HR 0.64, 95%CI 0.42–0.99, *p* = 0.048), but Insignificant in O vs. NW (HR 0.76, 95%CI 0.49–1.17, *p* = 0.211)	Insignificant in NW vs. OW vs. O	Insignificant in NW vs. OW vs. O	-	-	-	Insignificant in NW vs. OW vs. O	-
Hosseini et al. [32]	2023	USA	1176	WHO	-	-	Insignificant in UW vs. NW (OR 1.87, 95%CI 0.38–9.2), OW vs. NW (OR 1.03, 95%CI 0.45–2.31), class I obesity vs. NW (OR 1.07, 95%CI 0.45–2.55), class II obesity vs. NW (OR 1.06, 95%CI 0.35–3.21), class III obesity vs. NW (OR 2.19, 95%CI 0.83–5.76)	-	-	-	-	-
Iung et al. [130]	2014	France	3833	BMI categorized into <18.5, 18.5–29.9, ≥30 kg/m^2^ groups	Favorable all-cause 30-day mortality in BMI ≥ 30 vs. BMI (18.5–29.9) (OR 1.51, 95%CI 1.01–2.27, *p* = 0.047) and BMI ≥ 30 vs. BMI < 18.5 (OR 2.27, 95%CI 0.26–2.54, *p* < 0.001)	-	-	-	-	-	-	-
Kamga et al. [131]	2013	Belgium	30	BMI used as a continuous variable	Favorable all-cause 1-year mortality with 1 unit BMI increase (HR 0.54, 95%CI 0.35–0.85, *p* = 0.007)	-	-	-	-	-	-	-
Kische et al. [132]	2016	Germany	172	BMI categorized into <30 and ≥30 kg/m^2^ groups	Insignificant all-cause 1-year mortality in BMI < 30 vs. BMI ≥ 30 (93.1% vs. 83.2%, *p* = 0.06)	Insignificant in BMI < 30 vs. BMI ≥ 30	Insignificant in BMI < 30 vs. BMI ≥ 30	Insignificant in BMI < 30 vs. BMI ≥ 30	-	-	Insignificant in BMI < 30 vs. BMI ≥ 30	Insignificant in BMI < 30 vs. BMI ≥ 30
Kodali et al. [133]	2012	Multi-national	699	BMI used as a continuous variable	Favorable all-cause 1-year mortality with 1 unit BMI increase (HR 0.93, 95%CI 0.9–0.97, *p* < 0.001)	-	-	-	-	-	-	-
Koifman et al. [29]	2016	USA	491	Low BMI: ≤20; NW: 20.1–24.9; OW: 25–30; O: >30 kg/m^2^	Insignificant all-cause 1-year mortality in OW vs. NW (HR 1.21, 95%CI 0.69–2.11, *p* = 0.5) and O vs. NW (HR 0.87, 95%CI 0.47–1.64, *p* = 0.68)	Insignificant in Low BMI vs. NW vs. OW vs. O	Significant difference in Low BMI (20%) vs. NW (6%) vs. OW (11%) vs. O (8%) (*p* = 0.05)	Insignificant in Low BMI vs. NW vs. OW vs. O	-	Insignificant in Low BMI vs. NW vs. OW vs. O	Insignificant in Low BMI vs. NW vs. OW vs. O	Insignificant in Low BMI vs. NW vs. OW vs. O
Konigstein et al. [134]	2015	Israel	409	WHO, BMI used as continuous variable	Insignificant 30-day all-cause mortality in BMI ≥ 25 vs. BMI < 25 (OR 0.98, 95%CI 0.94–1.02, *p* = 0.4), but Favorable 1-year all-cause mortality in BMI ≥ 25 vs. BMI < 25 (OR 0.54, 95%CI 0.29–0.99, *p* = 0.048) Favorable long-term (mean follow-up 542 ± 390 days) all-cause mortality in BMI ≥ 25 vs. BMI < 25 (HR 0.6, 95%CI 0.37–0.97, *p* = 0.041) Favorable all-cause 1-year mortality with 1 unit BMI increase (HR 0.94, 95%CI 0.89–0.99, *p* = 0.043)	Insignificant in UW and NW vs. OW vs. O	Significant difference in UW and NW (7%) vs. OW (6%) vs. O (16%) (*p* = 0.013)	Insignificant in UW and NW vs. OW vs. O		Insignificant in UW and NW vs. OW vs. O	Insignificant in UW and NW vs. OW vs. O	Insignificant in UW and NW vs. OW vs. O
Luo et al. [135]	2022	China	109	Low: <21.9; Middle: 21.9–27; High: >27 kg/m^2^	Insignificant all-cause 35-month mortality in Middle vs. Low (HR 1.12, 95%CI 0.87–1.97, *p* = 0.5) and High vs. Low (HR 1.23, 95%CI 0.92–1.995, *p* = 74)	Insignificant in Low vs. Middle vs. High	Insignificant in Low vs. Middle vs. High	Insignificant in Low vs. Middle vs. High	Insignificant in Low vs. Middle vs. High		Insignificant in Low vs. Middle vs. High	Insignificant in Low vs. Middle vs. High
Maeda et al. [136]	2022	Japan	17,655	WHO	Favorable all-cause 1-year mortality in multivariate logistic regression using BMI category (UW, NW, OW, O) (HR 0.93, 95%CI 0.92–0.95, *p* < 0.0001)	-	-	-	-	-	-	-
Mancio et al. [103]	2017	Portugal	170	NW: <25; OW: 25–30; O: ≥30 kg/m^2^	Insignificant all-cause long-term [medical follow-up of 1.2 years (0.4–2.7)] mortality in NW vs. OW (HR 1.31, 95%CI 0.65–2.61) and O vs. OW (HR 1.43, 95%CI 0.67–3.06)	-	-	-	-	-	-	-
McInerney et al. [91]	2021	Europe, USA	3174	Nonobese: 18.5–29.9; Morbidly Obese (MO): ≥40 kg/m^2^ or ≥35 kg/m^2^ with obesity-related comorbidities ([99,137])	Insignificant in-hospital or 30-day mortality in Nonobese vs. MO (3.6% vs. 4.6%, *p* = 0.368) Insignificant long-term [median follow-up 14.1 months (6.5–36.0)] freedom from all-cause (79.4% vs. 80.6%, *p* = 0.731) and cardiovascular (88.7% vs. 87.4%, *p* = 0.699) mortality in Nonobese vs. MO.	Insignificant in Nonobese vs. MO	Significant difference in Nonobese (4.3%) vs. MO (6.6%) (*p* = 0.043)	Insignificant in Nonobese vs. MO	Insignificant in Nonobese vs. MO	-	Insignificant in Nonobese vs. MO	Insignificant in Nonobese vs. MO
Mok et al. [138]	2013	Canada	319	BMI used as continuous variable	Insignificant all-cause long-term [median follow-up 12 months (7–25)] mortality with each decrease by 1 log-transformed BMI unit (HR 1.05, 95%CI 1.00–1.10, *p* = 0.114)	-	-	-	-	-	-	-
Om et al. [139]	2019	Korea	379	BMI categorized into first tertile: ≤22.3; second tertile: 22.4–24.8; third tertile: ≥24.9 kg/m^2^	Favorable 1-year all-cause (19% vs. 10% vs. 6%, *p* = 0.006) and cardiovascular or stroke (19% vs. 13% vs. 6%, *p* = 0.01) mortality in BMI ≤ 22.3 vs. BMI 22.4–24.8 vs. BMI ≥ 24.9	Insignificant in BMI ≤ 22.3 vs. BMI 22.4–24.8 vs. BMI ≥ 24.9	Insignificant in BMI ≤ 22.3 vs. BMI 22.4–24.8 vs. BMI ≥ 24.9	-	-	-	Insignificant in BMI ≤ 22.3 vs. BMI 22.4–24.8 vs. BMI ≥ 24.9	Insignificant in BMI ≤ 22.3 vs. BMI 22.4–24.8 vs. BMI ≥ 24.9
Owais et al. [74]	2020	Germany	1609	WHO	Favorable all-cause 1-year mortality in BMI ≥ 25 vs. BMI < 25 (HR 0.36, 95%CI 0.21–0.6, *p* = 0.01)	Insignificant in NW vs. OW vs. O	Significant difference in NW (5%) vs. OW (7%) vs. O (18%) (*p* = 0.015)	Insignificant in NW vs. OW vs. O	-	Insignificant in NW vs. OW vs. O	Insignificant in NW vs. OW vs. O	Insignificant in NW vs. OW vs. O
Quine et al. [75]	2020	Australia	634	WHO	Favorable all-cause 2-year mortality in OW vs. NW (HR 0.56, 95%CI 0.38–0.85, *p* = 0.006), but Insignificant in O vs. NW (HR 0.71, 95%CI 0.46–1.08, *p* = 0.11)	Insignificant in NW vs. OW vs. O	Insignificant in NW vs. OW vs. O	Insignificant in NW vs. OW vs. O	Insignificant in NW vs. OW vs. O	-	Insignificant in NW vs. OW vs. O	-
Salizzoni et al. [140]	2016	Italy	1904	WHO	Favorable all-cause long-term [median follow-up 773 days (468–1126)] mortality in OW vs. NW (HR 0.78, 95%CI 0.62–0.97)	-	-	-	-	-	-	-
Seiffert et al. [141]	2013	Germany	326	BMI categorized into <20, 20–25, and >25 kg/m^2^ groups	Favorable all-cause 1-year mortality in BMI < 20 vs. BMI 20–25 (HR 3.2, 95%CI 1.52–6.76, *p* = 0.002), but Insignificant in BMI > 25 vs. BMI 20–25 (HR 0.86, 95%CI 0.5–1.47, *p* = 0.583)	-	-	-	-	-	-	-
Seiffert et al. [142]	2014	Germany	845	BMI used as continuous variable	Favorable all-cause 1-year mortality with 1 unit BMI increase (HR 0.96, 95%CI 0.93–0.99, *p* = 0.021)	-	-	-	-	-	-	-
Sgura et al. [143]	2022	Italy	794	UW: <20; NW: 20–24.9; OW and O: ≥25 kg/m^2^	Favorable all-cause 30-day mortality in BMI ≥ 25 vs. BMI < 25 (HR 0.33, 95%CI 0.13–0.84), and long-term (median follow-up 2.2 years) mortality in BMI ≥ 25 vs. BMI < 25 (HR 0.61, 95%CI 0.43–0.87)	-	-	-	-	-	-	-
Sharma et al. [23]	2020	USA	31,929	National Heart, Lung, and Blood institute criteria ^‡^	Favorable	Significant 30-day difference in UW (14.7%) vs. NW (13.8%) vs. OW (12.8%) vs. class I obesity (12.2%) vs. class II obesity (10.9%) vs. class III obesity (11.6%) (*p* = 0.013)	Insignificant in UW vs. NW vs. OW vs. class I obesity vs. class II obesity vs. class III obesity	Significant 30-day difference in UW (3.8%) vs. NW (2.8%) vs. OW (2.3%) vs. class I obesity (2.1%) vs. class II obesity (1.8%) vs. class III obesity (1.6%) (*p* = 0.01)	Insignificant in UW vs. NW vs. OW vs. class I obesity vs. class II obesity vs. class III obesity	-	Significant difference in UW (5.6%) vs. NW (7%) vs. OW (7.6%) vs. class I obesity (7.7%) vs. class II obesity (6.8%) vs. class III obesity (8.8%) (*p* = 0.024)	Insignificant in UW vs. NW vs. OW vs. class I obesity vs. class II obesity vs. class III obesity
Tezuka et al. [19]	2022	Japan	1693	WHO; UW: <18.5; NW: 18.5–25; OW: >25 kg/m^2^	Insignificant 30-day all-cause mortality difference in UW (1.7%) vs. NW (0.9%) vs. OW (0.3%) (*p* = 0.18) Favorable long-term [median follow-up 585 days (233–1029)] all-cause mortality in BMI < 18.5 vs. BMI ≥ 18.5 (HR 1.66, 95%CI 1.17–2.36, *p* = 0.005), cardiovascular mortality in BMI > 25 vs. BMI ≤25 (HR 0.46, 95%CI 0.23–0.94, *p* = 0.032), and non-cardiovascular mortality in BMI < 18.5 vs. BMI ≥ 18.5 (HR 1.78, 95%CI 1.16–2.75, *p* = 0.009)	Significantdifference in UW (3.7%) vs. NW (2.2%) vs. OW (0.7%) (*p* = 0.036)	Significant difference in UW (3.3%) vs. NW (2.9%) vs. OW (0.5%) (*p* = 0.018)	Insignificant in UW vs. NW vs. OW	Insignificant in UW vs. NW vs. OW	-	-	Insignificant in UW vs. NW vs. OW
Tokarek et al. [144]	2019	Poland	148	WHO, BMI used as continuous variable	Insignificant all-cause 30-day mortality difference in NW (16.2%) vs. OW (5.5%) vs. O (5.4%) (*p* = 0.15) Significant all-cause 1-year mortality difference in NW (32.4%) vs. OW (13.7%) vs. O (5.4%) (*p* = 0.03) Favorable all-cause 1-year mortality with 1 unit BMI increase (HR 0.91, 95%CI 0.85–0.98, *p* = 0.018)	Insignificant in NW vs. OW vs. O	-	Insignificant in NW vs. OW vs. O	Insignificant in NW vs. OW vs. O	Insignificant in NW vs. OW vs. O	Insignificant in NW vs. OW vs. O	Significant difference in NW (10.8%) vs. OW (1.4%) vs. O (8.1%) (*p* = 0.05)
Van der Boon et al. [28]	2013	Netherlands, Italy, France	940	WHO, BMI used as continuous variable	Insignificant all-cause 30-day mortality in OW (86.6%) vs. NW (87.4%) (*p* = 0.65) and NW (87.4%) vs. O (89.6%) (*p* = 0.72) Favorable all-cause 30-day mortality (HR 0.93, 95%CI 0.86–0.98, *p* = 0.023), but Insignificant long-term [median 12 months (6–18)] (HR 1.01, 95%CI 0.96–1.07, *p* = 0.73) mortality with 1 unit BMI increase	Insignificant in UW vs. NW vs. OW vs. O	Insignificant in UW vs. NW vs. OW vs. O	Insignificant in UW vs. NW vs. OW vs. O	Insignificant in UW vs. NW vs. OW vs. O	-	-	Insignificant in UW vs. NW vs. OW vs. O
Van Nieuwkerk et al. [89]	2021	USA, Brazil, Israel, Europe	12,381	WHO	Favorable all-cause 30-day mortality OW vs. NW (OR 0.73, 95%CI 0.61–0.88, *p* = 0.001), O vs. NW (OR 0.74, 95%CI 0.6–0.92, *p* = 0.006) Insignificant all-cause 30-day mortality in class II obesity vs. NW (OR 0.95, 95%CI 0.67–1.35, *p* = 0.79), and class III obesity vs. NW (OR 1.09, 95%CI 0.62–1.84, *p* = 0.76) Insignificant all-cause 1-year mortality OW vs. NW (HR 0.89, 95%CI 0.79–1.02, *p* = 0.09), O vs. NW (HR 0.95, 95%CI 0.82–1.1, *p* = 0.96)	Favorable in OW vs. NW (OR 0.74, 95%CI 0.59–0.94, *p* = 0.012), O vs. NW (OR 0.62, 95%CI 0.46–0.82, *p* = 0.001)	-	Insignificant in UW vs. NW vs. OW vs. O	Insignificant in UW vs. NW vs. OW vs. O	Insignificant in UW vs. NW vs. OW vs. O	Favorable in OW vs. NW (OR 1.3, 95%CI 1.1–1.53, *p* = 0.002), O vs. NW (OR 1.3, 95%CI 1.07–1.58, *p* = 0.009)	-
Wenaweser et al. [145]	2011	Switzerland	200	BMI categorized into <20, 20–25, and ≥25 kg/m^2^ groups	Favorable in all-cause 30-day mortality in BMI < 20 vs. BMI ≥ 25 (HR 6.6, 95%CI 1.48–29.5)	-	-	-	-	-	-	-
Yamamoto et al. [146]	2013	France	3072	WHO	Favorable all-cause 1-year mortality in OW vs. NW (HR 0.81, 95%CI 0.66–1, *p* = 0.05) and O vs. NW (HR 0.74, 95%CI 0.57–0.97, *p* = 0.029)	Insignificant in UW vs. NW vs. OW vs. O	Significant difference in UW (11.6%) vs. NW (4.4%) vs. OW (4.8%) vs. O (5.4%) (*p* = 0.018)	Insignificant in UW vs. NW vs. OW vs. O	Insignificant in UW vs. NW vs. OW vs. O	-	Insignificant in UW vs. NW vs. OW vs. O	-
Yamamoto et al. [147]	2015	France	777	BMI categorized into <20, 20–24.9, and ≥25 kg/m^2^ groups	Insignificant all-cause 30-day (89.9% vs. 84%, *p* = 0.37) and 1-year (71.1% vs. 60.3%, *p* = 0.54) mortality in BMI < 20 vs. BMI ≥ 20 Insignificant all-cause 30-day (89.9% vs. 88%, *p* = 0.76) and 1-year (73.1% vs. 65.1%, *p* = 0.61) mortality in BMI < 20 vs. BMI 20–24.9 Insignificant all-cause 30-day (91.4% vs. 97.9%, *p* = 0.17) and 1-year (71.4% vs. 82.7%, *p* = 0.33) mortality in BMI < 20 vs. BMI ≥ 25 Insignificant all-cause 30-day (93.8% vs. 90%, *p* = 0.67) and 1-year (87.5% vs. 75%, *p* = 0.36) mortality in BMI < 18.5 vs. BMI 18.5–20	Insignificant in BMI < 20 vs. 20–24.9 vs. ≥25	Insignificant in BMI < 20 vs. 20–24.9 vs. ≥25	-	Insignificant in BMI < 20 vs. 20–24.9 vs. ≥25	-	Insignificant in BMI < 20 vs. 20–24.9 vs. ≥25	Insignificant in BMI < 20 vs. 20–24.9 vs. ≥25

AF: atrial fibrillation; AKI: acute kidney injury; MI: myocardial infarction; NW: normal weight; O: obese; OW: overweight; PPM: pacemaker; UW: underweight. World Health Organization (WHO) BMI classification: underweight (≤18.4 kg/m^2^), normal weight (18.5–24.9 kg/m^2^), overweight (25–29.9 kg/m^2^), obese (≥30 kg/m^2^; class I: 30–34.9 kg/m^2^; class II: 35–39.9 kg/m^2^; class III or morbidly obese: ≥40 kg/m^2^); ^‡^ Same as WHO BMI criteria.
